# Trust, consistency and transparency: in-home respite needs and preferences of people living with dementia and their carers

**DOI:** 10.3389/frhs.2025.1550729

**Published:** 2025-07-08

**Authors:** Caroline Grogan, Sarah Harriman, Elizabeth Martin, Rebecca Waite, Olivia Fisher

**Affiliations:** ^1^ Health Services Research, Wesley Research Institute, Brisbane, QLD, Australia; ^2^ Faculty of Health, University of Queensland, Brisbane, QLD, Australia; ^3^ Faculty of Health, Charles Darwin University, Darwin, NT, Australia; ^4^ BlueCare, Brisbane, QLD, Australia

**Keywords:** dementia, respite, aged care, in-home respite, implementation science, family carers, health services research

## Abstract

**Purpose:**

To identify the needs, preferences, and perspectives of people living with dementia and their carers to inform design and implementation of an in-home respite service.

**Design/Methodology:**

Exploratory, interpretivist, pre- implementation qualitative study using Consolidated Framework for Implementation Research.

**Participants:**

People living with dementia and carers.

**Data collection:**

Multi-person and individual semi-structured interviews.

**Findings:**

15 participants: Four people living with dementia, 11 carers. Carers are exhausted and want a say in the development and delivery of services. People living with dementia and carers need safety, trust in respite staff and in the organisation, consistency, additional supports, and clear, transparent communication.

**Future directions:**

Findings will inform in-home dementia respite models of care, better supporting family carers and people living with dementia to age-in-place. Recommendations: provide an orientation session; clear, transparent communication; provide/refer carers to wrap-around supports; ensure consistency including having consistent carers, arrival times, services provided and routines; emergency and scheduled options.

## Introduction

Globally, there are 55 million people living with dementia (1). In 2023, it was estimated 401,300 Australians were living with dementia, two-thirds of whom were residing in the community, enabled by an estimated 137,600–354,200 carers (2). Older people and people living with dementia have a united wish to remain living in their own home (3). Informal family carers, hereafter referred to as “carers”, are pivotal in enabling this wish (4). Caring brings with it social, financial, and psychological burdens that can be profoundly impactful on carers' quality of life (4–7). To support carers in their caring role, regular short respite breaks are encouraged. The temporary relief of these burdens through respite care supports carers to maintain the caring relationship, and delay or avoid a transition into residential care for people living with dementia (4).

There are various models for planned or emergency respite breaks from caring duties, provided through family or friends or paid service providers. Respite can also be in the form of short breaks, supported holidays and leisure or art activities (8). In this study we focused on formal government-subsidised respite care. Formal paid respite options include community day centre-based, community-cottage, residential-nursing or in-home respite (9). Community centre-based respite is a group respite option where activities are facilitated at a community centre, with morning tea and lunch provided. Community-cottage respite is a house in the community where people can stay overnight for a few days or a week, and paid personal carers are rostered to support the older people. One house can support 3–4 people at a time. Residential-nursing respite is when the older person stays at an aged care facility, and in-home respite is when the worker comes and stays at the home of an older person (9).

Although respite services are widely available, they are frequently deemed by carers and people living with dementia to be inaccessible and not always appropriate to their needs (10, 11). Carers Australia's 2022 Carer Wellbeing Report indicated that of all Australian carers, those caring for a person living with dementia were most likely to access respite services (43.5% of all respite care) (5). However, the need for respite is likely much higher than its uptake. Carers report poor access to support and respite options including overnight in-home respite (80.6% of carers surveyed) (5). Vandepitte et al. (12) reported mixed outcomes from family carers and people living with dementia when they used residential respite, for instance lack of sleep for people living with dementia and distress for family carers. Furthermore, O'Shea et al. (13) found the acceptability of services was quite low.

Simply increasing the volume of respite care services is not enough to ensure that services are accessible and suitable. Access to support that appropriately meets the needs of people aligns with the World Health Organisation Global Action Plan on the Public Health Response to Dementia (14) and the United Nations Convention on the Rights of Persons with Disabilities (15). In previous studies carers have reported respite challenges which primarily fall into two main categories: access issues and unsuitable services. Access issues include high costs of services, long waiting times, complicated administration processes, lack of coordination between services, and at times explicit denial of a service to people living with dementia (5, 10, 11, 16). The recent Australian Royal Commission into Aged Care Quality and Safety also found respite options and accessibility limited (17, 18). Although respite can be helpful, it can also be disruptive, resulting in confusion or distress for people living with dementia (16, 19). Carers of people living with dementia have expressed a preference for in-home support (20). The familiarity and homeliness of a respite environment can reduce these negative consequences (16), yet the home environment can still hold challenges for people living with dementia and carers. In addition, Phillipson et al. (24) and Schirmer et al. (5) found carers' and people living with dementia's attitudinal barriers impacted their willingness and ability to access respite. For example, family carers' prior experiences influenced their perception of what respite would look like, often associating respite with an understaffed institutional setting (5). In-home respite is often preferred over other options such as day respite, cottage respite and nursing home respite (11, 21), particularly because the person living with dementia can stay in their trusted home environment (12). People living with dementia and their carers often find respite services unsuitable because they are often inflexible, and few adjustments are made for individual care needs, personalities, or preferences. Rather, a person is expected to fit into the service provider's schedule, e.g., medicine and food at set times (5, 19).

Limitations of respite care can be addressed by actively involving people living with dementia and their carers in the design and evaluation of respite services and acting on their recommendations thus addressing the Australian Royal Commission into Aged Care Safety and Quality's recommendation 1.3.c. “Enable people entitled to aged care to exercise choice and control in the planning and delivery of their care”; and 3aii) “putting older people first so that their preferences and needs drive the delivery of care” [ (17), p.206]. Carers often wish to be involved in service delivery design but lack the opportunity or awareness of how to do so (22). There is a growing body of evidence emphasising the importance and value of engaging people living with dementia in planning dementia care, although barriers are still experienced (23, 24).

To better deliver person-centred respite services, aged care providers need evidence-based guidance to inform the design and implementation of these services. Unfortunately, there are substantial evidence gaps. Research on respite services has primarily focused on residential and day respite programs (4, 25), with a lack of evidence for in-home services. Zhu et al.'s (25) systematic review of dementia service implementation studies identified a paucity of research on implementing respite services generally, and no previous pre-implementation research on in-home respite services for people living with dementia (25). Most studies have focused on intervention outcomes (25). This means there is a paucity of evidence explaining how and why outcomes were or were not achieved. Interventions delivered included new models of care in the form of electronic health platforms, psychoeducational workshops, day respite care, exercise and physical activities and care coordination and case management (25). Only two of these studies considered the needs and perspectives of carers and none of the studies incorporated the perspectives of people living with dementia (25).

Using implementation science-informed approaches and participatory service design and evaluation methods can improve service suitability for the users of that service (26, 27). The engagement and involvement of carers and people living with dementia in service design and provision is fundamental for improving acceptability and delivering care that meets their individual needs.

Implementation science research in dementia is growing but is still in nascent stages (25). Without the use of theoretical frameworks, studies are harder to generalise (28). By using an evidence-based implementation science framework such as the Consolidated Framework for Implementation Research (CFIR), implementation strategies and outcomes can be understood at a deeper level and generalisability is clearer for service providers (29, 30). This approach allows aged care organisations to understand the multiple dimensions of an intervention and identify what works when, where and why. The intentional inclusion of the voices of people living with dementia and their carers is essential in the design and implementation of services for people living with dementia to ensure they are fit-for-purpose. Without this, the current inflexible service models will likely continue to be norm and, whether intentionally or not, will continue to be unsuitable for people living with dementia and carers. In-home respite offers a flexible, preferable and familiar form of support (16, 20). The research team aimed to inform the development of an in-home respite service for people living with dementia and their carers based on their needs and preferences. This study contributes to in-home respite services by highlighting and amplifying what carers and potential clients want and need from service providers.

## Materials and methods

### Study design

The research team was approached by the service provider to conduct a qualitative pre-implementation study at 3 community aged care sites in Southeast Queensland. The service provider aimed to ensure that the design and implementation of a potential in-home respite service would best meet the needs of its users. Therefore, the scope of this study was to determine the preferences, needs and perspectives of people living with dementia and their carers for in-home respite services. Evaluation was outside of the scope of this study. An implementation science approach was interwoven throughout this study in the planning, data collection, analysis, reporting, and recommendations. This study is presented in line with the 32-item checklist Consolidated Criteria Reporting Qualitative Research (COREQ) (31) (see Supplementary Appendix A) and the Standards for Reporting Implementation Studies (32) (Supplementary Appendix B).

### Theoretical approach

Interpretivism was used to acknowledge how the same reality can be experienced differently by different people (33). This epistemological approach appreciates individuals' various experiences of the same phenomena. We coupled this approach with the use of implementation science theories, guided by the CFIR (29). This multi-theoretical framework is widely used for health service improvement. It draws on organisational, behavioral, and implementation theories and practices, and provides a guide to organisations looking to design, transform or evaluate new services. It has previously been used to guide implementation of aged care and dementia-focused services (25). The research team determined that the CFIR would be the most appropriate framework for this study because it was designed to identify pre-implementation determinants (barriers and enablers) to effectively design and implement health and aged care services. The CFIR has been extensively and successfully used to identify and address contextual influences in health and aged care implementation research.

### Setting

Three sites were included across Southeast Queensland, Australia. Regional site 1 at Beaudesert had 68 clients. Regional site 2 at Toowoomba had 250 clients and metropolitan site 3 at the Gold Coast had 401 clients. To note: not all service clients were eligible for the in-home respite service because of the level of approved subsidised government funded formal support they receive.

### Research team

The lead author has (CG) has more than 10 years' experience working in aged care community services. She brings insight into the aged care system and how to engage people living with dementia in research. CG conceptualised and developed the research project, conducted individual and multi-person interviews and data analysis. Senior author (OF) is an implementation scientist and has more than 20 years' experience in project management and research roles, guiding conceptualisation and design of the project. RW has over 17 years in aged care, managing implementation and delivery of state-wide community services. RWs knowledge of the service and managers at the sites aided in recruitment and project design. She has extensive experience, over 10 years across areas of nursing care and is highly skilled at working across disciplines. SH supported data generation in multi-person interviews and interpretation of data. EM is a health economist and implementation scientist with a background in mixed methods health services research, bringing insight into the data analysis process of the project. The collaborative team approach embedded reflection throughout the project, where positionality was discussed so personal assumptions did not overly influence data interpretation instead consensus was sought if disagreements occurred (34, 35). Interviewers and coders knew the CFIR well, and probing questions during the interviews were informed by their knowledge of CFIR constructs relevant to implementation of an aged care service.

### Recruitment strategy and participation

Participants were recruited through three locations, in Queensland, Australia. Purposive sampling was used to recruit potential clients of the service. Participants were invited to partake in a 30 min interview or an hour for multi-person interviews. The service provider was only able to support participant recruitment and data collection within a very limited timeframe of five weeks for all three sites. Therefore, the sample size was determined based on practical considerations rather than data saturation. Furthermore, RW was involved in linking the research team to the staff at each site those staff then identified possible research participants, she had no prior long-term interactions with possible research participants. The staff at each site identified existing clients who would be eligible for the new service and contacted them by phone or email to invite them to a multi-person or individual interview. The research team found that the aged care staff were only inviting a smaller subset of potential participants based on their own personal interpretation of who might be suitable, whether or not a potential participant met the study eligibility criteria. Subjective assumptions were made by the aged care staff, for example some people were deemed by staff as unlikely to access respite care and therefore they did not contact those potential participants. This reduced the number of potential and actual participants. This was realised during data collection at the second site so the process changed for the last site. The staff at the last site provided the research team with phone numbers or emails of potential participants. The research team then contacted potential participants directly, which improved overall numbers. Most participants did not know the research team prior to the interviews, unless they had been contacted directly by the research team (only one site) during the recruitment process. Participants participated in either individual or multi-person interviews.

Study eligibility:

1. Ability to make an informed decision.

AND

2. A person who met the following service eligibility criteria for the in-home respite service:
  People living with dementia or other conditions, illnesses, or injuries who may utilise the in-home respite service. The focus of the respite service was on supporting people living with dementia therefore that was the focus for this study, but people with other conditions were not excluded.

AND

3. Clients on a Home Care Package (Australian government funding to access community aged care services).

OR

4. Informal and unpaid carers (friends, family, or others) who regularly care for people living with dementia (or other conditions), who were eligible to access the Dementia In-Home Respite Service at the time of recruitment.

### Data collection

Multi-person and individual interviews were conducted between May 2023 and July 2023. Two members of the research team facilitated 1 face-to-face multi-person interview per site, 2 individual interviews with Gold Coast participants, 1 with a Beaudesert participant and 1 with a Toowoomba participant. Those who participated chose to have their carer present. Individual and multi-persons interview guides were informed by the CFIR (29). Please see Supplementary Appendix C and D for individual and group interview guides. Although the questions in the interview guide were framed in plain language, they related closely to CFIR constructs, particularly the “Individuals” domain (innovation recipients and deliverers), and the “Inner Setting” domain (work infrastructure/ organisational processes). Participants who were unable to attend the multi-person interviews at their local site, or had a preference for a 1 on 1 conversation, were offered the opportunity for a face-to-face, phone or online individual interview. There was one repeat interview: 1 carer who was interviewed over the phone wanted to share an additional experience she had remembered after her first interview. Therefore, another time was allocated for her to share further. Multi-person interviews were located at the aged care organisation's sites. The multi-person interview environment was flexible, relaxed and welcoming. The room was set-up with seats around large tables, with morning tea, coffee and tea provided. Individual interviews were conducted in participants' preferred locations, e.g., their own homes. We created a safe space by allowing time to build trust and rapport whilst ensuring participants knew they could take a break whenever they needed. Interviews were audio recorded, transcribed by a professional transcription service, and de-identified during data analysis. Unstructured field notes were handwritten after individual and multi-persons interviews. These detailed reflections on the interaction and non-verbal behaviour of participants (36).

### Ethics

Ethical approval was obtained from the UnitingCare Queensland Human Research Ethics Committee, approval number: 20230302. People living with dementia had the option to be interviewed with or without their family carer. Rigorous processes based on previous research with similar participant groups (37, 38) informed the process of assent, valuing the autonomy and right for people to be involved in research for altruistic and beneficence reasons. This was considered at the beginning and over the course of the research project, considering participants' verbal, behavioural and emotional forms of expression. Assent involves two key factors; the affirmative agreement to participate (can be verbal or non-verbal) and ability to make a meaningful decision. For example, someone who has read (or been read) the participant information document and able to understand the project, what their participation involves, understands the risks and benefits of their involvement and gives assent. This involves behavioural, emotional and verbal- indication of affirmative agreement to participate (37). No formal screening tool was used to assess participants’ decisional capacity, research has shown how using a tool can impact the rapport between the researcher and participant (39).

People living with dementia had the chance to review easy-read participant information and consent forms prior to the scheduled individual and multi-person interviews. This allowed time to ask questions about the project with the research team and talk with family members about their participation. People living with dementia also had the option to have a support person present in interviews. All people living with dementia who participated had a support person, namely their carer with them. We acknowledge how carers and people living with dementia can have differing views and perspectives but also see the value of respecting the choice of people living with dementia to have a support person with them during research. This aligns with the National Statement on Ethical Conduct in Human Research (40). Rolling informed consent was used throughout the project and interactions (38), where researchers monitor the nonverbal signs of assent and if signs are shown that they no longer wish to participate then this is respected and the interview is concluded, to either be continued after a break, rescheduled or simply concluded. No individual or multi-persons interviews needed to be stopped. Verbal consent was obtained by the carers who were interviewed over the phone (3).

### Data management and analysis

Multi-person and individual interviews were recorded and transcribed verbatim. Interviews ranged from 20 min to an hour with one participant reconnecting after the interview to share additional information. All multi-persons interviews were one hour. A combined deductive and inductive coding approach was used, with an apriori set of codes drawn from the Consolidated Framework for Implementation Research's constructs (29), followed by inductive derivation of codes and themes to detail the subtheme within the CFIR construct. Coding to a framework is frequently used within implementation science as a pragmatic qualitative data analysis strategy (34, 41). NVivo 11 was used to facilitate the coding process. Two of the research team individually coded all transcripts and field notes. Three (two of whom conducted data collection) then met regularly to discuss themes and ensure coding reliability and trustworthiness (42). When there were incongruent interpretations, discussions took place until agreement was reached. A fourth member of the research team with extensive experience in implementation science projects and coding to a framework provided guidance on the interpretation of CFIR domains and constructs. Initial coding was to the CFIR framework. Themes and subthemes were then thematically derived from the text coded to CFIR constructs, with some themes representing more than one CFIR domain or construct. For example, we created the theme “carers need a break” from the following subthemes: carers are tired and stressed; carers have unmet needs; and carers need regular and emergency respite. This theme was placed within the individual domain of CFIR. Due to the small sample size of each participant group (people living with dementia/ carers) at each site, data saturation was not considered to be reached within any single site. Nevertheless, data saturation of carer responses and the responses of people living with dementia were reached within the complete data set. Data saturation was considered to be reached when “no new codes” occurred in the data (43). This enabled the formation of rich, well defined descriptive themes.

Aged care practice recommendations were developed as part of the research by (1) using our review of literature described in the introduction of this paper to understand existing international and national policy guidance for aged care; (14, 15, 17). and (2) using these high-level policies and the expertise of our research team to translate our results into practical ways the participants' voices could be brought to life.

Following analysis and development of themes, all participants were phoned to see how they wished to receive the findings, over the phone, email or hard copy. A one-page summary of findings and recommendations written in accessible language was then shared to all participants (available upon request of the corresponding author). This was an information sharing activity to close the communication loop between researchers and participants. Participants did not query or disagree with any of the themes shared; there were no changes to findings from participants. Confirming the interpretation of data was not a one-off instance instead it was a continual process during interviews where clarification was sought and verified (44). Pseudonyms were used throughout presentation of results.

## Results

In total, eleven carers and 4 people living with dementia and 1 person with Parkinson's participated in this study. Ten carers were female, with 1 male. Of the 5 clients; 2 were females living with dementia, 2 were males living with dementia, and 1 was a male with Parkinson's Disease. No further demographic information was recorded. Multi-person interviews were conducted by CG and SH, whilst individual interviews were conducted by CG. It is not possible to report the response rate because the research team do not know how many people were contacted and declined to participate. We conducted 4 face- to-face multi-person interviews: 2 at the Gold Coast, 1 in Toowoomba and 1 in Beaudesert. Five semi-structured interviews were conducted over the phone (*n* = 3) or in-person (*n* = 2). Demographic information is displayed in Table 1.

**Table 1:** Participant demographic information.

Pseudonym	Role	Sex	Location	Interview type
Ben	Person living with dementia	Male	Beaudesert	Multi-person face-to-face
Moxley	Person living with dementia	Male	Gold Coast	Multi-person face-to-face
Melanie	Person living with dementia	Female	Gold Coast	Multi-person face to face
Brittany	Person living with dementia	Female	Toowoomba	Multi-person face to face
Brian	Person living with Parkinson's Disease	Male	Toowoomba	Multi-person face to face
Libby	Carer	Female	Gold Coast	Multi-person face-to-face
Lita	Carer	Female	Gold Coast	Multi-person face-to-face
Bella	Carer	Female	Gold Coast	Individual face-to-face
Carole	Carer	Female	Beaudesert	Individual phone
Cassandra	Carer	Female	Toowoomba	Multi-person face-to-face
Jeff	Carer	Male	Gold Coast	Multi-person face-to-face
Kira	Carer	Female	Gold Coast	Individual phone
Vicky	Carer	Female	Beaudesert	Multi-person face-to-face
Harris	Carer	Female	Toowoomba	Multi-person face-to-face
Trisha	Carer	Female	Toowoomba	Multi-person face-to-face
Herma	Carer	Female	Toowoomba	Individual phone

Four of these were individual interviews with carers.

Participants' responses focused heavily on a narrow set of CFIR domains and constructs that reflected their primary concerns and suggestions. No inductive codes were derived that did not represent one of the CFIR domains and constructs. Within and across domains, 4 overarching themes were identified. The themes are summarised in Table 2 and mapped to the corresponding CFIR domain or construct. In some cases, a theme addressed more than one CFIR domain or construct.

**Table 2:** Overarching themes mapped to consolidated framework for implementation research (CFIR) domains and constructs.

CFIR domain/construct	Results theme
IV Individuals domain	
Construct I: innovation recipients, individuals, need^a^	1. Carers need a break
	2. Carers and people living with dementia wish to be heard and involved in service delivery – *carers and people living with dementia need services that are appropriate for them, and being involved in service design and delivery can help to ensure these services are appropriate*
	3. Carers and people living with dementia need safety and trust in service providers
	4. Carers want transparency from service providers – *carers stated that they need to understand the services that will be provided, costs, scheduling and other practical considerations*
Construct H: innovation deliverers^b^	3. Carers and people living with dementia need safety and trust in service providers
	4. Carers want transparency from service providers *– participants felt that at times service providers can lack consistency with administration and costs and want greater transparency*
III Inner setting domain	
Construct A.3: work infrastructure & construct 3: communications^c^	4. Carers want transparency from service providers – *improved transparency and communication through improved work practices*

^a^ These themes relate to the expressed needs of innovation recipients (people living with dementia and carers).

^b^ These themes and subthemes related to people living with dementia and carers’ perspectives on what they want and need from the people delivering in-home respite care.

^c^ This theme related to the desire for service providers to have improved work infrastructure (processes) for communication to improve transparency.

Four key themes were identified:

1. Carers need a break.
2. Carers and people living with dementia wish to be heard and involved in the development and in service delivery.
3. Carers and people living with dementia need safety and trust in service providers at an individual and organisational level.
4. Carers want transparency - understanding administration, feedback channels and service costs.

Research has shown family carers and people living with dementia can and do have differing opinions (13). In this study we found no significant differences in opinions. It is possible that this was influenced by the fact that all people living with dementia chose to be interviewed with their carer and therefore may have been less likely to express disagreement. We acknowledge this as a potential limitation of this study. The concerns raised were similar across carers and people living with dementia. Some elaborated more about a specific concern, for example consistency of a worker, but the sentiment was reinforced by other participant groups. There were no differences of opinion between groups. There was little dissention around the topics participants shared. The only time there was disagreement was around the topic of trust, where some trusted the organisation, but others did not or trusted in one aspect (meeting care needs) and not another (administration).

### Theme 1 – carers need a break

Carers are exhausted and have emotional, physical and social needs which are sacrificed for the care and support of a person living with dementia. Carers consistently shared how they had little (if any) time for themselves. There was little time for carers to keep in touch with life-long friends, make doctor appointments or have a complete rest and recharge.

“I would love just for 24 h to, I can fly down in one day and fly back again, that sort of thing but it [the respite service] would have to be in the home.” (Libby, female carer)

“When was the last time that you had a break?” (interviewer) “Well, when I had that operation, that was my break.” (Lita, female carer)

“Would just like to nap and watch tv have a glass of red wine listen to music and rest.” (Bella, female carer)

“I think from a, a positive perspective what would be great is if we would be able to get away overnight.” (Carole, female carer)

The inability for carers to have a physical, emotional and mental break from their caring role was further displayed in the lack of time to socialise and connect with friends or activities they had previously enjoyed. For example, staying connected to book club.

Participants expressed that by not having opportunities to rest, their “worlds begin to shrink”. Maintaining hobbies and relationships takes time and commitment, with a caring role there are other priorities that take precedence and so connection with the wider community reduces significantly. Carers can become socially isolated in their caring roles:

“I was trying to go to book club. At that point in time he’d been very sick and really couldn’t be left alone.” (Cassandra, female carer)

“It’s a very … an alone position, you know where sometimes you think well, I’m on my own here and I’ve got to solve it and I think that’s, it’s stressful …” (Libby, female carer)

“Sometimes I have to cancel my own appointments so I can be on hand for my father’s appointments).” (Bella, female carer)

Carers expressed the feeling of being alone and isolated in their caring role. Carers drew attention to the struggle of maintaining social outings or even medical appointments. They are stressed and concerned about needing emergency respite. If a carer is isolated and something unforeseen happens to their own health (e.g., Stroke) then the person they care for can be left without support.

Both emergency and scheduled dementia in-home respite are needed. Carers shared how not knowing what will happen to the person living with dementia in an emergency played on their minds, so having emergency respite options along with scheduled breaks was a key consideration when choosing services.

“The hardest part for me in terms of working with an agency is sometimes it’s more the timing, if it’s urgent, that’s what’s important for me if there’s something I really urgently have to do… it would be nice to not have …the stress the whole time that he’s at home alone.” (Libby, female carer)

“From my experience… it’s more, almost emergency like when I, I had a stroke… a month ago and I was suddenly like… I had to sort of scramble to get my daughter to fly up… and my daughter had to take a week off work and move in with my husband.” (Jeff, male carer)

“When you need the help, the help is not there…when you deal with dementia you do not know when you need extra help” (Bella, female carer)

The need to have planned and emergency respite was a key consideration for carers wanting to access in-home respite. This key consideration links well with the second theme where voice and ability to contribute to service delivery design was raised.

### Theme 2 – carers and people living with dementia wish to be heard and involved in service delivery

Carers and people living with dementia wanted to be able to choose who entered their homes and receive real-time updates during the service. Carers and people living with dementia wanted to be asked and heard, to have their voices shape the service they accessed. Ben shared how being an introvert he would be very careful in who he allowed into his home, especially if they were to stay overnight.

“I think you would have to be very selective in the person that you sent to stay in my house and I would have to put up with overnight.” (Ben, male person living with dementia)

Jeff acknowledged how he would prefer the staff member to be someone he had met before and who knew the set-up of the house.

“And if, as we said before it would be nice if it was someone we’d met before who’s been before.. who’s familiar with the house yeah.” (Jeff, male carer)

Moxley highlighted how the final decision of who the staff member is and whether they are suitable or not should rest with him and his wife and not be imposed upon them by the organisation.

“And then they’ll do well and, you know, go forward if they’re happy with the person and trust them and it’s all those different words that mean a lot.” (Moxley, male person living with dementia)

Participants spoke about specific aspects of the service that were important to them. Their emphasis on this highlighted the need for services to respect and facilitate clients' autonomy and choice. Carers indicated that they want more autonomy over the service and the ability to choose staff. For instance, carers indicated a desire to meet staff before a service to see if they were the right fit and that personalities matched, they wished to be involved in all aspects of the service to truly co-design the service that they receive. Other areas for consideration were around the rostering of staff:

“…if you can have the one person basically spends the whole night or two nights… I don't know how from an industrial relations point of view how you’d get around that.” (Jeff, male carer)

Carers and people living with dementia wanted to be asked for their opinions or feedback, and listened to, to help design an appropriate service that is truly person-centred and meets their needs. Being able to voice their opinions openly involves a level of trust in service providers and staff.

### Theme 3 – carers and people living with dementia need safety and trust in service providers

Trust was a key consideration for carers and people living with dementia. This was twofold: trust in the organisation and trust in the staff. Some carers had absolute trust in the organisation while others were more skeptical. Kira shared how a friend of hers was being over charged for services, but how she trusted the staff at the organisation.

“… they were being ripped off, you know, whereas I could come here [to the organisation] and, I know I can trust them …” (Kira, female carer)

“I trust [organisation] and we’ve got everything under [the organisation], and we’ve found them very good.” (Lita, female carer)

[Referring to being able to trust a person with their personal care and in their home] “And to get the right people.” (Moxley, male person living with dementia)

Lita was very trusting of the service provider and found they listened and responded to her wishes, building more trust in the provider. Moxley equated trust to trusting the staff who come into his home. In contrast some carers felt they needed to keep a record of things because of a lack of trust and being charged in the past for services they had not received.

“I even take a photo now of the page I sign, so that I know that the service was done on that day because when I go back through my records, they might say, well, here’s a thing to say they’re coming, but sometimes they don’t front up at all, you know, and I just write it down now, so, I’ve got myself covered for the last two years. So, but that’s just an example of what can happen …” (Kira, female carer)

Mistrust and the need for trust was seen at the individual staff level and centred on the ability to have complaints answered. For Kira at the Gold Coast, there were two elements of trust, both of which were not stagnant, but changed as her experience with the service provider changed over time. For example in the above quote she became distrustful of the records and amount being charged for a service, so kept her own records, because she had been previously charged for a service that was cancelled. Below she then mentions how at first she was hesitant in leaving the house with a staff member alone with her mother, but as she got to know the staff member she then trusted them and could leave the house.

“I know I can trust them; you know. At first, I wouldn’t go out even, until I felt comfortable, you know.” (Kira, female carer)

“Now, you know, I can’t say, I can’t ring up here and say don’t send that girl here because.. Well, we did once and all hell broke loose, didn’t it?” (Vicky, female carer)

Vicky shared about a time when there was miscommunication between the staff member who visited them and an administration staff at the organisations office. When she raised a concern, the wrong information was shared with her and then there was a lack of follow-up. Now she is reluctant in raising anything with the service provider.

A perceived lack of quality of care can influence carers' reluctance to trust the next respite staff member. Trust and safety is needed in the delivery of services and care standards must be met.

“Some of them [aged care staff] just gave up too quickly and there was one lady who had her in and out of that shower, I took the dog outside, up this hill, I was gone 10 min and she was out and dressed in that time. There’s no way she gave her a shower and I go in and you could see hardly any water on the base of the chair, you know, around the wheels. She’d obviously just wet the washer and given her a, as they say, top and tail …I’d say to them … if she’s hitting you or being annoying, call me and I will assist you in getting that nightie off.” (Kira, female carer)

Kira shared a hesitancy of leaving her mother with a staff member. When she did leave for a short amount of time the personal care service of supporting her mother to have a shower was not adequately done. This then created a mistrust towards a staff member, which relates closely to carers not being able to have a break (Theme 1) because they feel they have to always be available. Participants emphasised that trust is developed when personal routines and preferences are respected. Carers wanted the staff to know their family member living with dementia and be flexible to their needs. This involves the need to create a person-centred, flexible care plan. By acknowledging the routines of people, trust can be established between service providers and carers.

“… you shouldn't try and be too domineering or somehow, so you know obviously making suggestions about what to do and is… something which if Melanie doesn’t show any interest, it’s probably best just to drop it ..”(Jeff, male carer)

“… someone that would just go with the flow and not try and gee him up and or try and make him go for the walk and what have you because that, it’s just not going to get us anywhere and I think, so you put someone who is just quietly go with the flow, follow, he just has no interests at the moment at all, he’s lost interest in everything, so board games or whatever, even putting on the TV, he’d just say no… so someone who will just go and not try and fight that too much.” (Libby, female carer)

Wants staff with initiative and rapport with her father- “knowing when to step in and help and when to step back” (Bella, female carer)

“It would be good if he could have the same person who is a suitable person.” (Brittany, female carer)

“But I'll say I guess just that they would be a, an outgoing pleasant personality, be competent and reliable. I don't think age has got much to do with it, like one of dad’s carers is quite young, but she’s probably the best one that he has, and he’s established a really good rapport with her. So, it’s more so personality I think, and being competent.” (Carole, female carer)

The personality traits, attitude and intuition of staff were all mentioned as attributes a staff member should have. Carers valued the staff members ability to work with a person living with dementia and be flexible to their needs or wishes on a certain day. This flexible approach was also reflected by people living with dementia.

People living with dementia also raised the importance of understanding them and tailoring the service to their personalities, being flexible to their needs, and, not forcing something onto them.

“You don’t want a boxer after you. Yeah. Yeah, some people like go and, you know, that can be a problem. Most people are pretty great.” (Moxley, male person living with dementia)

“Yeah. And, I love people, I just love people. And, I’m, I’m, all my life I’ve, and it makes me, I have a bit of, but I know now that this is happening I, if anything happened, I, I would like to, I love people and dogs. You know. I love people. But I’m coming to the point where I may lose my, my communications… So I just love everyone…” (Brittany, female person living with dementia)

“the right person would have to be empathetic. Yeah. And I guess that’s really up, yeah, I think most people would do that, most people but I think it would have to be the right person” (Ben, male person living with dementia)

People living with dementia raised the need for staff to be responsive to their personalities, for instance Brittany loves company and talking and Moxley valued his independence and autonomy so did not want to be ordered around to do certain things by staff. Therefore, matching the right staff to a person would be important to have a successful respite service experience.

### Theme 4 – carers want transparency from service providers

There was confusion and anxiety around administration and costs. The confusion about costs and lack of transparency can create a lack of trust in service providers. Carers experienced stress and anxiety about the copious amount of paperwork involved in accessing services and support. Cassandra shared how she relied heavily on her daughter to help her navigate the paperwork involved with accessing the services she is currently receiving through the organisation.

“We sat down one day with daughter, and we were having trouble with… the [Commonwealth Home Support Program], for me … It was an absolute nightmare …” (Cassandra, female carer)

Carole described how working full-time and arranging her father's care was unsustainable and she was planning for some form of respite but felt overwhelmed when looking through all of the information and forms.

“… it’s such a minefield of paperwork to get them into outside respite … But … I was just reading through the paperwork last night and it does seem to be quite overwhelming.” (Carole, female carer)

The cost of the service was a concern for carers, with many acknowledging the potentially expensive new service.

“… providing their money in their package will cover it, because it wouldn’t be cheap.” (Vicky, female carer)

“It’d be very expensive though, you’d imagine.” (Lita, female carer)

“Cost is a big one, even though you’re on, he’s care plans… well going on the sheet we got with all the costs, it’s really high (cost) stuff. (Trisha, female carer).

“Very expensive to have someone come to the home compared with going to the facility but I know that’s what Harris would prefer … Certainly your package wouldn’t pay for, if it was, happen to be a week or a fortnight, I don’t think … It’s so much a day.” (Brittany, female carer)

The burden of administration and navigating the aged care system was a stress point for many carers. The cost involved in the respite service was a key consideration for carers' willingness to access the service which was a potential barrier to accessing in-home respite support.

## Discussion

In this qualitative study we used an interpretivist implementation science approach to hear and represent the voices of people living with dementia and their carers in service design and implementation, addressing a notable gap in existing literature. Four overarching themes were present in the data:

1. Carers need a break.
2. Carers and people living with dementia wish to be heard and involved in the development and delivery of services in service delivery.
3. Carers and people living with dementia need safety and trust in service providers at an individual and organisational level.
4. Carers want transparency - understanding administration, feedback channels and service costs.

The research team contends that addressing these four themes and associated subthemes in the development and delivery of in-home respite services is likely to improve the suitability, acceptability and uptake of these services by people living with dementia and their carers.

Carers need a break from their caring duties but there are important barriers that need to be addressed, and these align with the findings of previous research. For instance, preconceptions about what respite is can be a barrier (5, 24). Although this research highlighted that well-designed and delivered in-home respite services appeal to both people living with dementia and their carers, allowing a new person into their home presents risks and creates anxiety for both carers and people living with dementia (10, 45).

To provide a person-centred service, people living with dementia and family carers' perspectives need to be sought (46). Involving people living with dementia and their carers in the design and delivery of services for their use has the potential to improve the uptake, trust in, and suitability of those services (23). O'Shea et al. (21), reported an implementation gap regarding person-centred care. Active involvement in the design and delivery of in-home respite services can support people living with dementia and their carers to feel heard, with the intention of both developing trust in the service provider, but also to ensure that services meet their needs (23, 24). However, although carers and people living with dementia want to be involved in the design and delivery of services, this can be challenging. It is not enough to just ask for feedback, e.g., via a survey. Authentic involvement means an interactive and ongoing process of feedback provided in a way that suits clients, not just the service provider (24). Trust and relationships play an important role in whether and what information clients are willing to share with a service (47). People living with dementia and carers who have important information to share can sometimes decline to give feedback directly to a service due to concerns about what will happen as a result of their feedback. In this study, a client-carer dyad described what they perceived to be a negative experience following a previous complaint which meant they decided not to provide feedback to the service again in future. A recent study also found that sometimes clients and carers do not want to “bother” the aged care provider so they adapt to the service offered instead (22). Having systematic and interactive processes for people living with dementia and their carers to participate in service design and evaluation can help to traverse these issues (22).

The four overarching themes represent a strong need for trust, safety, transparency and clear communication, to enable carers to feel that they can take the break they need. Efforts need to be made by service providers to develop trusting relationships and a sense of safety. People living with dementia and their carers need to trust and have confidence in the quality of care provided by a respite service (10, 22, 45, 48). Carers' perception of and trust in the quality-of-care influences whether they choose to access respite care (19). The act of building a trusting relationship between clients and providers improves the quality of care received (22). Carers still have concerns about the quality of care received and training of staff (49, 50). Training staff in dementia care can improve services and increase staff confidence in delivering competent care (17, 48).

Aged care services tend to be difficult to navigate, inconsistent, and often lack transparency. It is well documented that the aged care systems internationally are complicated, unclear and administratively burdensome (16, 17, 49, 51). In this study, carers and people living with dementia expressed frustrations with what they considered opaque processes and a lack of consistency, e.g., with billing. They were also frustrated by what they perceived as a lack of communication about other practical considerations such as scheduling of services. Inconsistent timing was described as stressful, reflecting the findings of previous research (4, 9). Positive communication processes between respite staff, people living with dementia and carers is foundational for quality services. Without clear and timely communication, the quality of care is hindered (48, 50). Flexibility in respite services is important (4, 9), e.g., respecting a client's preferred schedule or recreational activities. However, carers and clients often see services as predetermined, with little flexibility (22). Shanley (52) called for services to embed a flexibility framework into practice to provide more creative and appropriate support. Knowing a client and family through consistent care is necessary to be able to provide an informed flexible approach and a quality support service (9). Continuity of care has been shown to have positive effects on staff and clients and contributes to higher levels of quality of care (53, 54). Inconsistency of care negatively influences a service's quality and client outcomes (48, 50), whereas familiarity between staff and clients has been linked with better continuity of care outcomes, increased wellbeing and a reduction of stress and anxiety for people living with dementia (50). Providing care navigation and administrative support is an opportunity for aged care providers to ensure that their clients are receiving the most appropriate supports.

This study confirms that carers need more than just respite support. The strain of caring can be substantial, and a rising need for emergency respite is indicative of an unmet need (16). Although caring impacts both the physical and emotional state of carers (4), emotional strain is often a greater challenge than physical strain (6), pointing to the need to support carers beyond just respite care. Schirmer et al (5). described how carers’ wellbeing was significantly higher if they were able to access respite, financial support, support from social networks and fellow carers. O'Shea et al. (21) similarly report respite alone is not an adequate service aim. Providing or facilitating carers to access wrap around supports (e.g., counselling, dementia training, peer-to-peer carer support) can help them to maintain their caring role.

The 2022 version of the CFIR provided a useful guide to the development of the interview guide and the framework analysis. Data in this study were narrowly focused, primarily on the “Individuals” domain (innovation recipients and innovation deliverers), with some discussion of “Outer” and “Inner” domain constructs such as local conditions and communication. This aligned with the expectations of the research team that people living with dementia and their carers would focus their responses primarily on their personal experiences as recipients or potential recipients of respite care. Although the CFIR framework was originally conceptualised for use in health services research (55), the CFIR constructs are also relevant for aged care services and provide a frame of overarching constructs within which more detailed subthemes can be placed. To our knowledge CFIR has not been used widely for in-home respite studies, only one other study (25) has previously used CFIR in aged care. Therefore, this study builds on the application of implementation science frameworks to guide aged care service delivery. Implementation scientists champion the importance of inclusive practices and hearing the voices of all relevant stakeholders, but people living with dementia typically continue to be excluded from implementation science studies (25). We acknowledge that the methods used to include the voices of people living with dementia in this study are not new but have been largely absent from implementation science research.

The recommendations presented in Table 3 provide practical guidance to aged care providers on considerations that need to be addressed. Each recommendation maps to one or more of the CFIR constructs and themes identified in this research. Because of the strong focus of people living with dementia and their carers in this study on development of trust and safety, we recommend taking an evidence-based approach to building this trust prior to commencement of services. Thus, our key recommendation is to invest in orientation and relationship building prior to provision of respite sessions. This provides the opportunity to develop rapport, trust and transparency, making people living with dementia and their carers feel more comfortable and confident in the service (47). Better understanding the client as an individual also enables the flexibility necessary to provide effective services, reflecting theme 4, the need for greater transparency.

**Table 3:** Recommendations, rationale, and alignment with key policies.

Recommendation and example in action	Results theme	Rationale	WHO global action plan on the public health response to dementia (14).	UN convention on the rights of persons with disabilities (15).	Royal commission recommendation (17).
1) Provide orientation sessions for staff and carers, regardless of whether the client and staff member have met previously. Example: Orientation sessions allow staff, carers and clients to plan activities, normal routine, and priorities.	Theme 2 – Carers and people living with dementia wish to be heard and involved in service delivery Theme 3: Carers and people living with dementia need safety and trust in service providers - personal routines and preferences need to be respected	Carers wanted respite staff to: • Know them and the person living with dementia. • Know their hobbies, interests, elements of life (e.g., farmer, grandfather). • Know how to support them in a non-obtrusive and flexible way (e.g., follow the client's lead, not pressuring them) • Be non-judgemental and match worker to client's personality	Action Areas 4 and 5	Article 9	1.3.c. 3.a.ii 3.b.x. 13.1.a 13.1.b. 13.1.c. 13.1.d. 13.1.e. 13.2
2) Provide time and opportunity for the voices of clients and PLWD to have direct input into service provision so the service is tailored, consistent and flexible to their needs. Ensure consistency and continuity. Example: Plans updated continually and collaboratively with carers and respite staff.	Theme 1: Carers need a break - Both emergency and scheduled dementia in-home respite are needed Theme 2 – Carers and people living with dementia wish to be heard and involved in service delivery Theme 3: Carers and people living with dementia need safety and trust in service providers - personal routines and preferences need to be respected	Carers wanted tailored and consistent support: • Support their routine and preferences. • Provided by the same respite staff (or team of staff). • Respite staff to have comprehensive understanding of clients’ care plans. • Have in-built flexibility. • Wanted a consistent service.	Action area 4	Article 19	2.b.iii 3.a.i. 3.a.ii
3) Provide direct, timely and clear communication, including feedback. Example: Having a case manager responsible for a client and act as the main point of contact.	Theme 3 – Carers and people living with dementia need safety and trust in service providers Theme 4: Carers want transparency from service providers	Carers spoke of times in the past when negative feedback was poorly managed, resulting in a reluctance to provide any further feedback. Conversely, when feedback was acted upon, and the carer was informed of the outcome this was seen positively.	Action Areas 4 and 5	Article 9	1.3.e. 2.b.v.
4) Be transparent and clear about administration processes and costs. Example: Review the layout, wording, and content of invoices and forms in partnership with clients to enhance clarity and transparency.	Theme 4: Carers want transparency from service providers	A lack of clarity on invoices, intake forms, and other documentation creates concern and anxiety amongst carers.	Action Area 6	Article 3	27.
5) Provide real-time updates of care being provided by respite staff for carers. Example: Updates via mobile application e.g., after showering, if there are any difficulties encountered.	Theme 3 – Carers and people living with dementia need safety and trust in service providers Theme 3 – Carers and people living with dementia need safety and trust in service providers - are standards must be met - personal routines and preferences need to be respected Theme 4 – Carers want transparency from service providers	Carers had concerns about leaving their loved ones in respite. Carers wanted real time updates about their loved one during the respite session. This could be in the form of a text or photo of the client while the carer is away.	Action Areas 6 and 7	Article 9	3.b.xvi.
6) Provide, or facilitate referrals to, appropriate supports for carers. Example: Refer carers to, or provide information about, existing supports- including emergency respite.	Theme 1 – Carers need a break Subtheme 1a: Carers’ worlds are shrinking -both emergency and scheduled dementia in-home respite are needed Theme 3 – Carers and people living with dementia need safety and trust in service providers - care standards must be met	Carers’ needs included emotional, practical, social, and educational needs. They found it hard to schedule and keep medical appointments or participate in social activities. Carers shared how there was a lot of trial and error in supporting PLWD and how they would have liked more information and education sooner, as well as to share experiences in a supportive environment with other carers. Family carers were concerned about what would happen if they experienced an emergency. Access to emergency respite is pivotal in supporting family carers.	Action Areas 1 and 5	Articles 3 and 9	15.1.a 15.1.b. 15.1.c. 15.1.d.
7) Provide a reliable and trustworthy service centred on clients’ needs. Example: Prioritise being a relationally focused service, building trust and rapport between the organisation, staff members, carers and clients.	Theme 3 – Carers and people living with dementia need safety and trust in service providers – care standards must be met	Carers and PLWD need to be able to trust and rely on the respite service. Carers wanted a service they could trust. Distrust occurred when a service was scheduled and then no one arrived, or when the carer did not trust the staff member enough to leave the house.	Action Area 4	Articles 5 and 25	32.a. 32.b.

### Recommendations

We intended to directly inform and influence practice, to ensure the service meets the needs, preferences and perspectives of people living with dementia and their carers. In Table 3, we outline seven recommendations for service providers wanting to develop a dementia in-home respite service. Our recommendations have been mapped to the results themes and aligned with recommendations from international aged care policies.

### Implications for research and practice

This study demonstrated that not only is it possible for people living with dementia to effectively, authentically and safely participate in implementation science research, they can provide important contributions to improve the suitability and acceptability of services intended for their use. The research team recommends using similarly inclusive methods to engage people living with dementia and their carers in continuous improvement and evaluation of health and aged care services. The recommendations in Table 3, although specific to the local context, can provide a guide for aged care providers developing an in-home respite service so it meets the needs of people living with dementia and their carers. The CFIR was an appropriate framework for use in the aged care setting.

### Limitations

Participants were limited to those who were eligible to access the new service. This meant only those who had an active home care package through the aged care provider were invited to participate. The lack of diversity of participants from culturally and linguistic diverse backgrounds was also a limitation. We did not record the cultural and linguistic background of participants because it was not the focus of the study, however, due to the researchers' time spent with participants they became aware of the limited diversity amongst participants. However, participant group sizes do not prevent significant insights in qualitative research (56). This study was specific to the Queensland, Australia context, however the use of a theory-informed implementation science framework enables comparison between studies and settings. This study focused solely on the perspectives of people living with dementia and their carers so although some domains and constructs from CFIR were relevant for this study, additional data from service providers and other sources would have been needed to fully address all CFIR domains and constructs.

### Summary

Hearing the voices of people living with dementia and their carers in the design of dementia in-home respite services will help to ensure that the service meets their needs and preferences. This is valuable for both service providers, who want high uptake of services and for people living with dementia and their carers, who want to be able to trust and rely on services. We have offered practical strategies to ensure that people living with dementia and their carers have their needs addressed in service delivery. Implementing these recommendations will better enable carers to have a break, support their wellbeing, and maintain the caring relationship long-term.

## Data Availability

The raw data is not available due to the sensitivity and identifiable nature. Further queries can be directed to the corresponding author.
