# Supplementary material for: Trust, consistency and transparency: in-home respite needs and preferences of people living with dementia and their carers
Source: Front Health Serv. 2025 Jul 8;5:1550729. doi: 10.3389/frhs.2025.1550729 (PMC12279863; doi:10.3389/frhs.2025.1550729)
Supplement: Supplementary file 1 [file Supplementaryfile1.docx]

**Appendix A Supplementary Material**

**Consolidated Criteria for Reporting Qualitative Studies (COREQ) Checklist**

*Table S1. Completed COREQ 32-item checklist for transparent and complete reporting of methods*

| **COREQ checklist** | **Location where item is reported** |
| --- | --- |
| **Domain 1: Research team and reflexivity** | |
| *Personal Characteristics* |  |
| 1. Interviewer/facilitator | Methods |
| 2. Credentials | Methods |
| 3. Occupation | Methods |
| 4. Gender | Methods |
| 5. Experience and training | Methods |
| *Relationship with participants* |  |
| 6. Relationship established | Methods |
| 7. Participant knowledge of the interviewer | Methods |
| 8. Interviewer characteristics | Methods |
| **Domain 2: study design** |  |
| *Theoretical framework* |  |
| 9. Methodological orientation and theory | Theoretical approach |
| *Participant selection* |  |
| 10. Sampling | Methods – Recruitment Strategy and participation |
| 11. Method of approach | Methods - Recruitment Strategy and participation |
| 12. Sample size | Methods - Recruitment Strategy and participation |
| 13. Non-participation | Methods - Recruitment Strategy and participation |
| *Setting* |  |
| 14. Setting of data collection | Methods |
| 15. Presence of non-participants | Methods |
| 16. Description of sample | Methods - Recruitment Strategy and participation. No demographic information was collected regarding year of diagnosis, time in caring role, age or gender. We did not record cultural and linguistic background of participants because it not the focus of the study, however, due to the researchers' time spent with participants they became aware of the limited diversity amongst participants. |
| *Data collection* |  |
| 17. Interview guide | Interview and group interview guides provided as supplementary material.  Some questions included; Any difficulties currently experienced? Ideas around them?  What would you like respite to look like?  What skills and qualities do you think staff need? |
| 18. Repeat interviews | One repeat interview, carer wanted to add some more information |
| 19. Audio/visual recording | Audio recorded |
| 20. Field notes | Recorded throughout data collection |
| 21. Duration | Methods |
| 22. Data saturation | Recruitment strategy and participation and Data analysis |
| 23. Transcripts returned | Methods |
| **Domain 3: analysis and findings** |  |
| *Data analysis* |  |
| 24. Number of data coders | Methods - Data analysis |
| 25. Description of the coding tree | Methods - Data analysis |
| 26. Derivation of themes | Methods - Data analysis |
| 27. Software | Methods - Data analysis |
| 28. Participant checking | Methods |
| *Reporting* |  |
| 29. Quotations presented | Yes in findings |
| 30. Data and findings consistent | Yes in findings |
| 31. Clarity of major themes | Yes in findings |
| 32. Clarity of minor themes | Yes in findings |
