# Supplementary material for: Trust, consistency and transparency: in-home respite needs and preferences of people living with dementia and their carers
Source: Front Health Serv. 2025 Jul 8;5:1550729. doi: 10.3389/frhs.2025.1550729 (PMC12279863; doi:10.3389/frhs.2025.1550729)
Supplement: Supplementary file 3 [file Supplementaryfile3.docx]

semi-structured interview questions

For people not comfortable with, or not able to participate in a focus group setting and who request a one-on-one interview

**Before the interview**

| ​​☐​ | Check if the participant has had a chance to review the participant information form, and ask if they have any questions prior to the interview |
| --- | --- |
| ​​☐​ | Explain that the questions relate solely to their experience in accessing in-home respite with the orgnisation and no questions will be asked about a person’s health status or medical history |
| ​​☐​ | Explain that the interview will be audio recorded and check if the participant agrees to being recorded |
| ​​☐​ | Confirm the expected interview duration with the participant and check whether they have a time they need to finish by, e.g., for another appointment |
| ​​☐​ | Let the participant know that they will have an opportunity at the end of the interview to add anything they would like to, and they are welcome to add any additional thoughts within two weeks of the interview by contacting the interviewer – additional information can be provided by email, phone or video conference as per the participant’s preference. |
| ​​☐​ | ***Commence audio recording*** |
| ​​☐​ | ***Obtain verbal consent to participate in the interview*** |

Interviews will be conducted in a semi-structured format. The wording of individual questions may change according to the participant’s role, and probing questions will be asked as needed to gain further information about key points. The overall topics and aims will remain the same.

Introduction:

- Welcome, meet and greet. Check if participant would like a drink or anything before we begin (if face to face).
- Research processes: Outline to participants (people living with dementia and informal carers) that interviews seek to gather perspectives of people living with dementia and their carers in the Ipswich and Toowoomba regions to help explore and understand the new care model to help improve it
- Describe how the data will be used and kept confidential
- Remind participants they can stop and have a break at any time

**Interview Questions**

*Understanding previous experiences*

- What would be your ideal experience of accessing respite?
- Have you used respite options with the organisation before?
  - What was that like?
  - What would make you feel more comfortable in accessing respite?
  - Would you prefer in-home respite? Why?
  - Would you change anything?

*Understanding needs*

- What practical considerations need to be taken into account?
- Do you have any concerns that need to be addressed?

*What’s missing?*

- Is there anything you would like to add/share further?

**At the end of the interview**

| ​​☐​ | Thank the participant for their time and feedback and will be invited to a thank-you lunch to show appreciation (after results are finalised) |
| --- | --- |
| ​​☐​ | Remind the participant that they are welcome to clarify their statements or add any additional information by contacting the interviewer by email within two weeks following the interview – their comments will be added to the transcript |
| ​​☐​ | ***Upload the audio recording to the server and delete from recording device*** |
