# Supplementary material for: Trust, consistency and transparency: in-home respite needs and preferences of people living with dementia and their carers
Source: Front Health Serv. 2025 Jul 8;5:1550729. doi: 10.3389/frhs.2025.1550729 (PMC12279863; doi:10.3389/frhs.2025.1550729)
Supplement: Supplementary file 4 [file Supplementaryfile4.docx]

semi-structured Multi-person INTERVIEWS Outline

# Introduction:

- Welcome
- Research processes: Outline to participants (people living with dementia and informal carers) that the focus group is aiming to:
  - Identifying the needs, preference and perspectives of people living with dementia and their carers in the Ipswich and Toowoomba regions
  - Identifying additional local contextual factors, such as the perspectives and preferences of staff in the Ipswich and Toowoomba regions that may influence the effectiveness of the In-Home Respite Service model of care
  - Making adaptations to the model of care prior to roll-out of the service in the Ipswich and Toowoomba regions
- Participants will be reminded that participation is voluntary and the session will be recorded.
- Describe how the data will be used and kept confidential
- You are free to take a break if you need
- Only have to share what you are comfortable with
- We will collect any written group activity notes that the groups are willing to share. Just leave them on the tables. Ensure that everyone has provided consent. Opportunity to ask questions.
- Introduce research team
- Housekeeping – e.g., location of bathrooms, finishing time

# First discussion as a whole group:

- Discuss people’s experiences of respite and their wants and needs of an in-home respite program
- Any difficulties currently experienced? Ideas around them?
- What would you like respite to look like?
- What skills and qualities do you think staff need?
- Is there anything else you would like to share?

# Activity one: Split group into carers and then people with dementia (where possible)

- This will allow each group to share personal feelings that they may not feel comfortable in saying in front of informal carer or in front of person they care for.
- Research staff to facilitate group activity on butcher paper of things people would like from respite including:
  - What would make you more comfortable in using it?
  - Pros and cons
  - Barriers to being able to use respite care
  - Concerns and how these could be addressed
- Butcher paper to be collected and kept for data analysis
- Add any additional components that the participants believe are missing

# Thank you and conclusion

- Option to provide further feedback by email to research team.
- Invite participants to leave contact details if they are interested in providing written feedback or would like to be involved in a one on one (face to face or virtual) interview, or to have their contact details added to the distribution list for the report.
- Outline next steps of the project.
